# Reflections on the surge in malaria cases after unprecedented flooding in Pakistan—A commentary

**DOI:** 10.1002/hsr2.1620

**Published:** 2023-10-10

**Authors:** Shehroze Tabassum, Tuaseen Kalsoom, Zaofashan Zaheer, Aroma Naeem, Ahmed Afifi, Laya Ohadi

**Affiliations:** ^1^ King Edward Medical University Lahore Pakistan; ^2^ Benha University Faculty of Medicine Banha Egypt; ^3^ Shahid Beheshti University of Medical Sciences Tehran Iran

**Keywords:** COVID‐19, infectious disease, malaria, public health

## Abstract

**Background:**

Malaria is a parasitic infection primarily caused by four main species of the genus *Plasmodium*, that is, *Plasmodium falciparum*, *Plasmodium ovale*, *Plasmodium vivax*, and *Plasmodium malariae*. It is transmitted through the bite of the female *Anopheles* mosquito. It holds the status of one of the leading causes of death in the developing world. Malaria is endemic to Pakistan, and the country experienced the worst floods in its history from April to October 2022. The stagnant flood water served as a breeding ground for mosquitoes, culminating in an alarming spike in malaria cases. According to the World Health Organization (WHO), the number of cases reported till August 2022 was more than in the whole year of 2021. There was more than a twofold rise in cumulative cases in 62 high‐burden Pakistani Districts in August 2022 as compared to August 2021.

**Aims:**

This commentary aims to bring this emerging issue to notice and highlight the most effective probable measures to help eliminate and prevent the hazards the current outbreak poses.

**Results:**

Rapid planning and execution are needed to ensure the most efficient and rapid elimination of malaria. To educate the general public, the national government must start public awareness efforts in electronic, print, and social media and deploy solar‐powered mobile healthcare units to far‐flung areas. Prophylactic and postexposure treatments should be planned because larvicidal preventive measures are less practical in flood‐affected vicinities.

**Conclusion:**

The most effective preventive strategy is drug prophylaxis, followed by insecticide‐treated nets, indoor residual spraying, and untreated nets. Scientists should intensify their investigations for effective medications to alleviate the malaria burden in Pakistan.

## 1 INTRODUCTION

Malaria, an infectious disease caused by the parasites of the genus *Plasmodium* and transmitted through the bite of the female *Anopheles* mosquito, holds the status of one of the leading causes of death in several developing countries of the world. Eighty‐seven countries and territories, housing nearly half of the world's population, have been deemed areas at risk of malaria transmission. Two billion people, including travelers and residents of endemic countries, are at risk of getting malaria, with 1.5–2.7 million deaths reported annually.^1^, ^2^

Five *Plasmodium* species have been implicated in malaria in human beings namely *Plasmodium falciparum*, *Plasmodium ovale*, *Plasmodium vivax*, and *Plasmodium malariae*, and the more recent *Plasmodium knowlesi*.^3^ Ninety percent of the world's malaria mortality and 99.7% of malaria cases have been attributed to *P. falciparum*, earning the reputation as the most prevalent and pathogenic specie of the malarial parasite.^4^, ^5^

**Figure 1:**
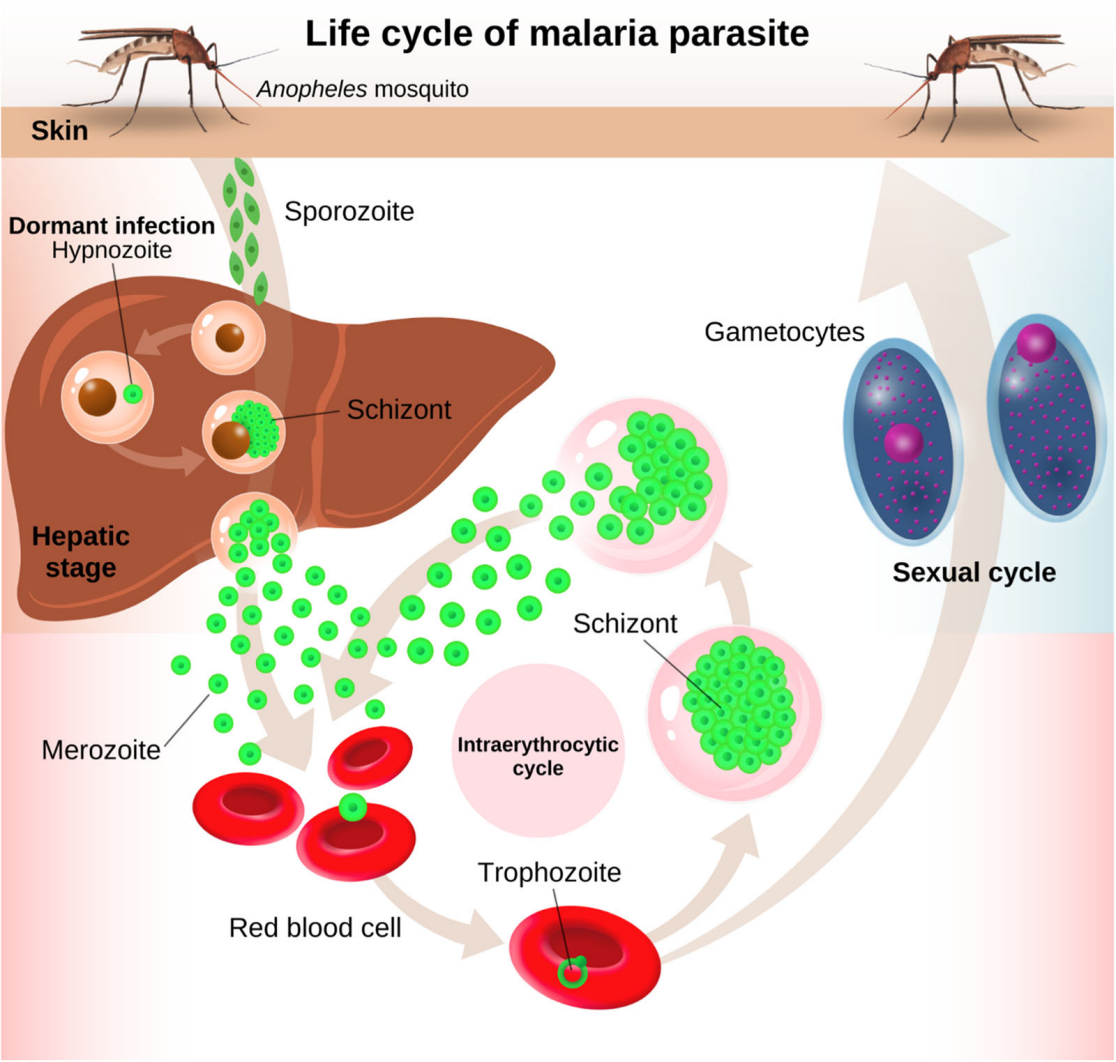
Life cycle of malaria parasite.

Malaria transmission involves the female *Anopheles* mosquito taking a blood meal from a person with nontreated malaria. This causes the ingestion of red blood cells containing male and female gametocytes by the mosquito, which are transformed into sporozoites upon the completion of their developmental process in the mosquito's gut and transported to its salivary glands. Inoculation of sporozoites, contained in the salivary glands into a capillary at the puncture wound of a noninfected person then follows.^6^ The malarial parasites initially make their way to the liver for maturation and upon the completion, they are released into the bloodstream. It is during this stage that signs and symptoms of malaria appear.^5^, ^7^ Life cycle of malaria parasite is illustrated in Figure 1.

The mean incubation period for *P. falciparum* is 12 days, and symptom presentation in endemic areas begins in the first or second month after the mosquito bite. Typically, one can expect to see signs and symptoms within a few weeks. These primarily include fevers, chills, headaches, nausea, vomiting, diarrhea, abdominal pain, fatigue, muscle weakness, joint pain, tachycardia, rapid respiratory rate, and cough.^8^ These nonspecific signs and symptoms of uncomplicated malaria often make diagnosis difficult. One percent to two percent of *P. falciparum* cases often lead to severe malaria, hallmark features of which include prostration, acidotic breathing, impaired consciousness, multiple convulsions, pulmonary edema, disseminated intravascular coagulation, acute kidney injury, jaundice, shock, and coma.^5^, ^9^, ^10^ High‐risk populations for malaria primarily comprise people with little or no immunity against the infection (children, pregnant women) and travelers to endemic areas. All species of Plasmodium, when contracted early in pregnancy, cause abortion or enhance the risk of neonatal death as a result of intrauterine growth restriction and prematurity.^11^ Other modes of transmission for malaria include transplacental and blood transfusion‐mediated transmission, these especially pose a problem for healthcare workers in non‐endemic areas.^8^

Clinical diagnosis of malaria based on signs and symptoms, and physical findings upon examination, is the traditionally employed method. Laboratory diagnostic methods like conventional microscopic diagnosis by staining thin and thick peripheral blood smears, other concentration techniques, including quantitative buffy coat (QBC) method, rapid diagnostic tests (ParaScreen, SD Bioline, Para check) are highly sensitive and specific while molecular diagnostic methods, such as polymerase chain reaction, loop‐mediated isothermal amplification, microarray, mass spectrometry, and flow cytometric assay techniques are even more, opening up new avenues for effective and rapid diagnosis and contributing to better clinical outcomes by permitting timely initiation of treatment.^12^

## 2 STATISTICS

Pakistan is a developing country, endemic to malaria. Climate change, melting of glaciers, and torrential monsoon rainfall, all contributed to the devastating floods in the country, which started back in June 2022.^13^ The havoc wreaked by the floods, the likes of which had never been seen before in the country, amounted to a death toll of 1700, with 12,867 people injured and 7.9 million people temporarily displaced as of October 2022.^14^

With no roof over their heads and a place to call home, these refugees are currently seeking shelter in temporary camps set up by the government. Living out in the open has exposed them to a number of vector‐borne diseases including malaria.^15^

**Figure 2:**
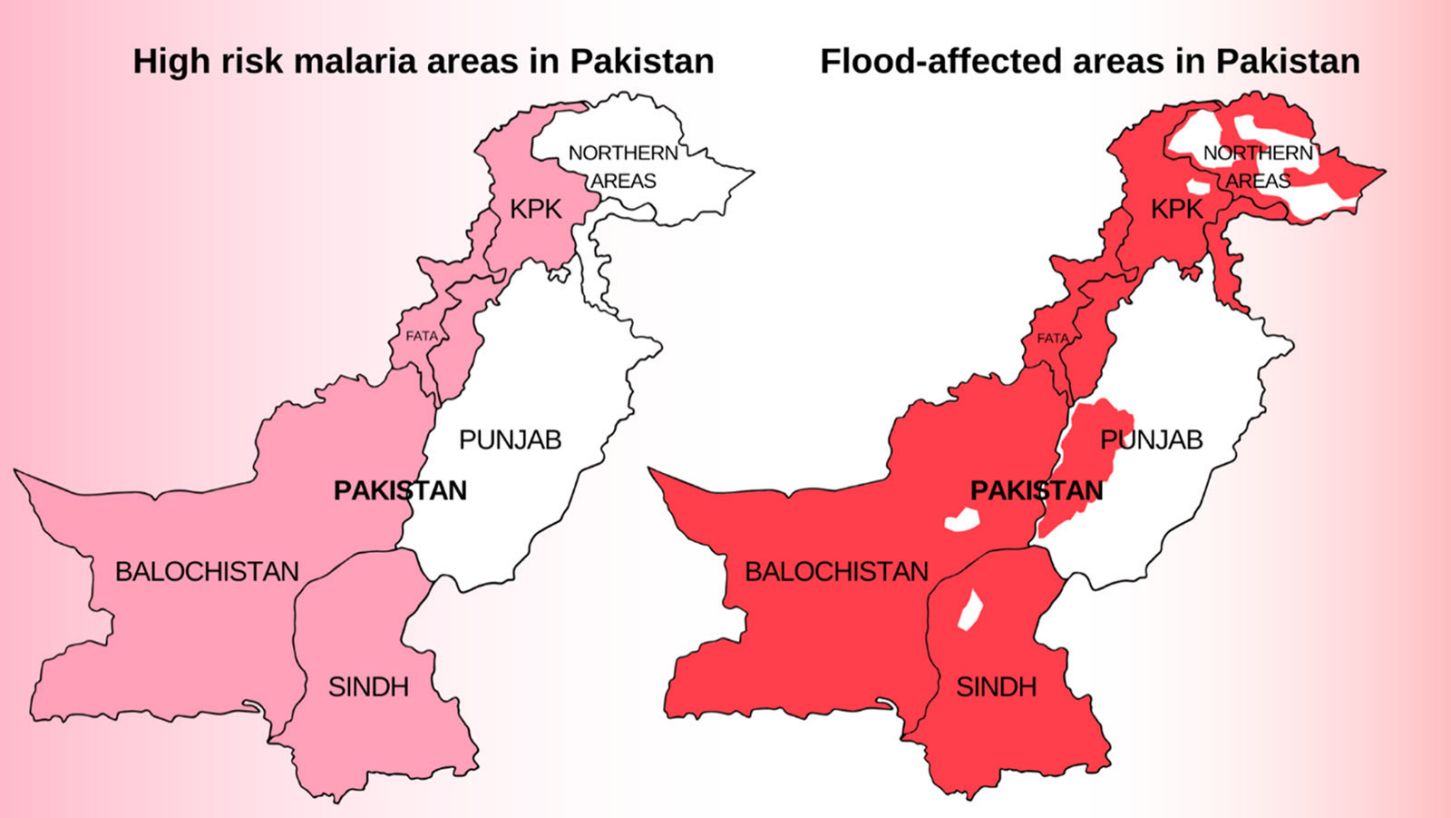
High‐risk malaria regions and flood‐affected regions in Pakistan.

The stagnant flood waters, covering vast expanses of land have served as breeding grounds for mosquitoes and the number of malaria cases in these flood‐affected regions, mainly Sindh and Balochistan, have skyrocketed.^16^ Figure 2 illustrates Pakistan's high‐risk malaria areas and flood‐affected areas in 2022.

**Figure 3:**
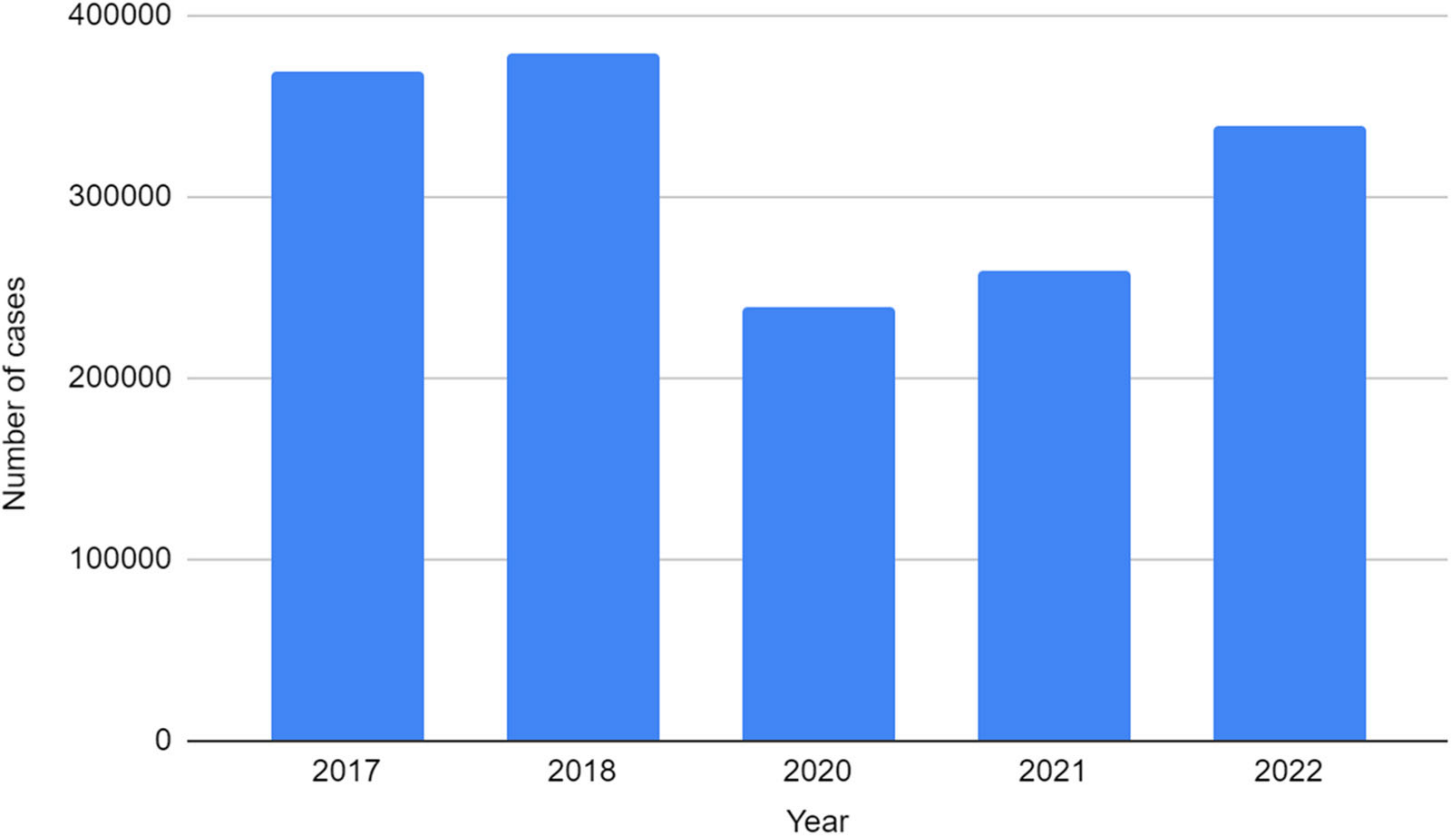
Number of malaria cases in Pakistan during the last 5 years till September 2022.

According to the World Health Organization (WHO), more malaria cases were reported till August 2022 than in the whole year of 2021 combined. The current malaria outbreak has been predominantly attributed to the *P. vivax* species. A huge disparity exists between the current malaria cases in Sindh province, Balochistan province, and high‐burden districts; and those that were reported in these regions at the same time last year. The number of confirmed malaria cases in Sindh province in August 2022 was 69,123 compared to 19,826 confirmed cases in August 2021. The number of confirmed cases in Balochistan province saw an increase from 22,032 in August 2021 to 41,368 in August 2022. The number of cumulative cases in 62 high‐burden Pakistani districts was recorded at 389,372 in September 2022 as compared to 178,657 cases in August 2022.^17^ Figure 3 illustrates malaria cases in Pakistan during the last 5 years.

## 3 CHALLENGES

Recent flooding in Pakistan has put the country in a health crisis more severer than one would assume. The toll of cases for this deadly disease may rise to 2.7 million, WHO warns^18^ unless extreme measures are undertaken on an urgent basis to combat the foe. However, there are multiple challenges the country is facing in this context. The post‐flooding influx has put the already tumbling healthcare infrastructure of Pakistan in shambles. The sudden emergence of malaria followed by a failure to timely implement the malaria control program led to a huge upsurge in patients. This rapid increase in malaria cases also resulted in a massive lack of medications, which made the people's woes worse.^19^ Considering how huge this outbreak of malaria is and the damage which has been done, a major economic investment is needed to condone the loss which cannot be borne by the country's poor economy alone at the moment. An Emergency Response was mobilized by WHO, national authorities, and humanitarian associations but according to reports, only 15%–20% of the affected population has been reached so far.^20^ The inability to reach the far‐off areas, the lack of rapid diagnostic kits at places, and thus the failure to keep an accurate track of actual cases became another striking issue. Rural communities mainly suffered from this havoc due to substandard living and hygiene conditions, and are the target audience of culminating efforts. But the prevalent illiteracy in these communities and general noncompliant attitude has been key issue. On the policymakers' part, insufficient resource allocation, ineffective training of the prescribers on accepted treatment recommendations, and poor integration of the malaria control program and its outreach stand as major hurdles in the way to eliminate malaria.^21^ The overlapping signs and symptoms of malaria with other viral and bacterial diseases also pose a great challenge in accurate and timely diagnosis. Table 1 provides comprehensive features of differential diagnosis of malaria.

**Table 1:** Features of differential diagnosis of malaria.

Sr. No.	Differential diagnosis	Causative organism	Signs and symptoms	Laboratory diagnosis	Mechanism of transmission
1	Brucellosis	Brucella (Gram‐negative zoonotic bacterium)	Undulating fever, night sweats, anorexia, osteomyelitis, hepatosplenomegaly	Serological testing, isolation, and slide agglutination testing.	Consumption of unpasteurized dairy products, direct contact with farm animals
2	Dengue	Filovirus (virus)	Fever, chills, skin rashes, facial flushing, headache, myalgias, arthralgias, and cervical lymphadenopathy	PCR, serological tests for an increase in antibody titer	Bite of female aedes mosquito
3	Typhoid fever	Salmonella Typhi (Gram‐negative bacterium)	Fever, rose‐colored maculae, pea soup diarrhea, hepatosplenomegaly	Lactose fermentation, cultures on hektoin agar, TSI agar tests, and cell cultures	Feco‐oral route
4	Measles	Paramyxovirus	Cough, coryza, conjunctivitis, koplik spots	PCR, increase in serum antibody titer and clinical diagnosis	Respiratory droplets

The Government of Pakistan, in collaboration with WHO did initiate the mass execution of indoor residual spraying (IRS) procedures and mass distribution of insecticide‐treated mosquito nets, however, this too is problematic in two contexts. First, the current revenue is not capable of catering to the existent dire need. Secondly, the more prevalent (>80%) form of malaria in current upsurge is due to the *P. vivax* species,^17^ which is notoriously less responsive to insecticide sprays and other preventive procedures‐owing to the reason that it usually bites in the mornings, and in outdoor settings.^22^ *P. vivax* malaria is more difficult to identify since there are often fewer parasites circulating in the blood of an infected person than there are with *P. falciparum* malaria. Moreover, the existing diagnostic methods cannot detect the dormant liver hypnozoite type early in the course of an infection. *P. vivax* gametocytes can hence spread even before an infection is recognized or treated, posing a risk of uncontrolled transmission.^23^ The existing diagnostic methods, hence, cannot detect the dormant liver hypnozoite type.^24^ In addition, the only approved drug for *P. vivax* infections, primaquine causes mild to severe hemolysis and anemia in patients with G6PD deficiency whose percentage of incidence is extremely high in the South Asia region and it highly limits the management of malaria in Pakistan.^25^

## 4 RECOMMENDATIONS

To ensure the elimination of malaria in the most effective and rapid way, swift planning and execution are required in multiple domains. A new effective strategy is required for Pakistan's malaria response program that should involve significant financial investment in systems for disease surveillance and outbreak response. Different iterations of existing systems are currently all financed by donors and are not a top concern for Pakistani policymakers, which definitely needs to be looked into.^26^ Major economical support and funding from international sources are deemed essential in this situation. Public awareness campaigns must be launched by the government via electronic, print, and social media to educate the masses. Significant steps have to be taken to improve health infrastructure and outreach in far‐flung areas. Swift vector surveillance in all areas should be ensured, along with ensuring the widespread availability of rapid diagnostic testing units based on histological parasite confirmation. Solar‐powered mobile healthcare units must be deployed by the health department quickly to improve the reach and efficacy of these measures.^27^

Since larvicidal preventive procedures are not as feasible in flood‐affected areas, prophylactic treatment, and postexposure treatments should be planned.^28^ Considering the contraindication of the only approved drug for *P. vivax* infections, primaquine in G6PD deficient patients in areas like Pakistan, G6PD deficiency screening must be included in the long‐term treatment plans, while currently focusing more on preventive strategies and supportive treatment alternatives.

Owing to the difficulty in diagnosing currently prevalent *P. vivax* species, a considerable amount of further research is the need of the hour to develop more precise diagnostic methods. As transmission is likely to occur even before detection, timely measures must be enforced for prevention. Drugs for prophylaxis have been found to be the most effective preventive method followed by insecticide‐treated nets, IRS, and untreated nets.^29^ Gearing up the pursuit of highly‐effective prophylactic drugs is another recommendation for scientists.

In the times to come, effective planning and implementation of economic and healthcare reforms, along with continued efforts to eliminate poverty, improve living conditions and execute the country's response against climate change^30^ are expected to yield fruitful results.

## AUTHOR CONTRIBUTIONS

**Shehroze Tabassum**: Conceptualization; writing—original draft; writing—review and editing. **Tuaseen Kalsoom**: Writing—original draft. **Zaofashan Zaheer**: Writing—original draft. **Aroma Naeem**: Writing—original draft; writing—review and editing. **Ahmed Afifi**: Writing—original draft. **Laya Ohadi**: Writing—original draft.

## CONFLICT OF INTEREST STATEMENT

The authors declare no conflict of interest.

## TRANSPARENCY STATEMENT

The lead author Laya Ohadi affirms that this manuscript is an honest, accurate, and transparent account of the study being reported; that no important aspects of the study have been omitted; and that any discrepancies from the study as planned (and, if relevant, registered) have been explained.

## Data Availability

Data available on request from the authors.
